# Immune Checkpoint Inhibitors Related to Cardiotoxicity

**DOI:** 10.3390/jcdd9110378

**Published:** 2022-11-03

**Authors:** Ru Chen, Min Zhou, Feng Zhu

**Affiliations:** 1 Department of Cardiology, Union Hospital, Tongji Medical College, Huazhong University of Science and Technology, Wuhan 430022, China; 2 Clinic Center of Human Gene Research, Union Hospital, Tongji Medical College, Huazhong University of Science and Technology, Wuhan 430022, China; 3 Department of Pulmonary and Critical Care Medicine, Tongji Hospital, Tongji Medical College, Huazhong University of Science and Technology, Wuhan 430022, China; 4 Key Laboratory of Respiratory Diseases, National Ministry of Health of the People’s Republic of China and National Clinical Research Center for Respiratory Disease, Wuhan 430022, China

**Keywords:** immune checkpoint inhibitors, immune-related adverse effects, cardiotoxicity, myocarditis, cardio-oncology

## Abstract

Immune checkpoint inhibitors (ICIs) have now emerged as a mainstay of treatment for various cancers. Along with development of ICIs, immune-related adverse effects (irAEs) have aroused wide attention. The cardiac irAE, one of the rare but potentially fatal effects, have been reported recently. However, the clinical comprehension of cardiac irAEs remains limited and guidelines are inadequate for cardio-oncologists to tackle the problem. In this review, we have summarized current classifications of, manifestations of, potential mechanisms of, and treatment for ICI-related myocardial injury in order to provide some clues for the understanding of cardiac irAEs in clinical work.

## 1. Introduction

Immune checkpoint inhibitors (ICIs), a class of antibodies that amplify T-cell-mediated immune responses against cancer cells, have revolutionized cancer therapy. Immune checkpoints are inhibitory pathways processed by immune cells to regulate immune responses, and they are important pathways for tumor immune escape. Among all the immune checkpoints, cytotoxic T lymphocyte-associated antigen 4 (CTLA-4) and programmed cell death 1 (PD-1) and its ligand PD-L1 have been approved for clinical use. CTLA-4 competes with the co-stimulatory receptor CD28 for CD80 and CD86, thus inhibiting T cell activation [1]. In peripheral tissues, CTLA-4 also inhibits effector T cells by promoting the immunosuppression function of Tregs [2]. In contrast to CTLA-4, PD-1 inhibits the activation of T cells through cell-intrinsic signaling and restraining immunosuppression by Tregs [3,4]. Until now, more than 20 ICIs have been studied in over 800 clinical trials [5]. CTLA-4 antibodies (ipilimumab), PD-1 antibodies (nivolumab, pembrolizumab, cemiplimab, etc.), and PD-L1 antibodies (atezolizumab, durvalumab, avelumab, etc.) have been widely used. Candonilimab, a bispecific antibody targeting PD-1/CTLA-4, is now in clinical trials at different stages for several cancer types, including locally advanced unresectable or metastatic gastric adenocarcinoma or gastroesophageal junction adenocarcinoma (NCT05008783), advanced hepatocellular carcinoma (NCT04728321), and peripheral T cells lymphoma (NCT04444141).

Various cancer types have been approved for ICI treatment, including melanoma, non-small cell lung cancer (NSCLC), renal cell carcinoma, colorectal cancer, breast cancer, etc. [6]. Compared with chemotherapy, ICIs are associated with higher levels of quality of life and longer times to clinical deterioration [7]. It is expected that the indications for ICIs in cancer will continue to expand in the coming years [8]. What accompanies the prominent antitumor responses is unspecific organ damages known as immune-related adverse events (irAEs). Common irAEs include dermatologic, hepatic, gastrointestinal, and endocrine toxicities [9]. Most irAEs are reversible and respond well to corticosteroids; however, fatal irAEs have been noted with an incidence of 0.3% to 1.3% [10]. Compared with other irAEs, ICI-related myocarditis has the highest fatality rate of approximately 39.7%, although the incidence of myocarditis is estimated to be only 0.04–1.14% [11,12]. Apart from myocarditis, ICI-related cardiac injuries also include pericarditis, arrhythmias, and vasculitis, with varied symptoms, signs, and prognoses.

The global burden of disease is shifting from communicable disease to non-communicable disease and has included cancer since 1990 [13], creating increasing challenges for cardiologists and oncologists. Inadequate experience with ICI-related cardiotoxicity causes great hinderances in clinical work for oncologists and cardiologists. In this review, we aimed to summarize the current understanding of ICI-related myocardial injuries with regard to their epidemiology, potential mechanisms, clinical presentations, and management in order to raise common awareness of ICI-related cardiotoxicity.

## 2. Epidemiology

Some marketed ICIs worldwide are listed in Table 1. The incidence of ICI-related myocarditis ranges from 0.04% to 1.14%; however, its mortality (25–50%) is significantly higher than other irAEs, according to current publications [14,15]. Exponentially increasing cases of ICI-related myocarditis were reported from 2013 to 2018 [11]. Other cardiovascular toxicities have also been reported, such as acute coronary disease, pericardial disease, and vasculitis. Among all the cardiac irAEs, myocarditis is likely the most common early cardiac impairment, followed by pericardial diseases and conduction diseases [16]. Late cardiac adverse events are primarily heart failure with reduced ejection fraction (HFrEF) [17]. Hu et al. revealed that in clinical trials regarding lung cancer treated with PD-1 and PD-L1 inhibitors, the incidence of heart failure and cardiac arrest was 2% and 1%, respectively [18]. In a retrospective study, 4/60 (6.7%) patients with non-small cell lung cancer developed pericardial effusion during ICI therapy [19]. In a systemic review, Mir, H. et al. reported that approximately 10% of ICI-related cardiotoxicity cases developed cardiac conduction defects and 5–10% developed ventricular arrhythmias [20]. In addition, an ICI-induced inflammatory response might increase the risk of atherosclerosis [21]. It was reported that ICIs were associated with a three-fold higher risk for atherosclerotic cardiovascular events and a >three-fold higher rate of aortic plaque progression [22]. Mahmood et al. reported that major adverse cardiovascular events were observed in 0.52% of patients with ICI-related myocarditis [14]. In a registry involving 103 ICI-related myocarditis patients, 40% of the cases developed at least one major adverse cardiovascular event [23]. Escudier et al. reported a case fatality rate of 27% in 30 patients with ICI cardiotoxicity, including left ventricular (LV) systolic dysfunction, a Takotsubo-like syndrome, and arrhythmia, in addition to myocarditis [24]. The incidence of major adverse cardiovascular event (MACE) was been reported to be 10.3% during a median follow-up of 13 months in patients treated with ICI [25]. For Asian users of ICI, MACE occurred with an incidence rate (IR) of 1.7 per 100 patient-years; the IR of cardiovascular hospitalization was 5.6 [4.6, 6.9] episodes per 100 person-years, with 52.9 [39.8, 70.3] days’ stay per 100 person-years [26]. The prime publications about cardiotoxicity related to immune checkpoint inhibitors from 2018–2022 are listed in Table 2.

The risk factors for ICI-related cardiotoxicity are unclear. Patients who received combination ICI therapy had a higher incidence of myocarditis than those who received a monotherapy [51]. However, in a systematic review and meta-analysis that enrolled cancer patients, no significant difference regarding cardiac irAEs was found between single ICI and dual ICI therapies [45]. The overreporting of myocarditis in patients treated with anti-PD-1 vs. anti-CTLA-4 was noted [11]. By using the US Food and Drug Administration’s (FDA) Adverse Event Reporting System (FAERS) database, Yoshito Zamami et al. enrolled 13,096 cases that received five different ICIs and found that myocarditis risk was significantly higher in female patients (OR, 1.92; 95% CI, 1.24–2.97; *p*  =  0.004) and patients who were 75 years or older (OR, 7.61; 95% CI, 4.29–13.50; *p*  <  0.001) [52]. In addition, they also reported that a combination of ICIs (ipilimumab and nivolumab) was associated with an increased risk of myocarditis [52]. The incidence of ICI-related myocarditis does not differ among cancer types and among patients with preexisting cardiovascular disease [14]. Similarly, using univariate regression analysis, Dal’bo N. identified that pre-existing coronary artery disease and atrial fibrillation were not risk factors for myocarditis [53]. Conversely, Rushin P. Patel reported that patients with ICI-related myocarditis had a higher prevalence of cardiovascular risk factors, including hypertension, and were more likely to be on statin and angiotensin-converting enzyme inhibitors/angiotensin receptor blockers (ACEI/ARB) therapy [54]. In another retrospective study, individuals with previous heart failure and acute coronary syndrome were more likely to develop ICI-related myocarditis [55]. Underlying autoimmune disease and diabetes mellitus might be risk factors for ICI-associated myocarditis [56]. Genetic variation has been demonstrated to be associated with hypothyroidism irAE during atezolizumab treatment [57]; however, whether cardiac irAEs are affected by genetic variations remains unknown. In a cohort of 75 enrolled melanoma patients treated with anti-CTLA-4, anti-PD-1, or their combination, Michael F. Gowen et al. tested a baseline serum autoantibody before ICI therapy and identified that the antigen targets for toxicity-associated autoantibodies were significantly enriched in organs affected by irAEs [58]. In another cohort that enrolled 60 melanoma patients, fewer differentially expressed autoantibodies at baseline were detected in patients who experienced specific irAE compared with those who did not [59]. Whether cardiac-specific antibodies could serve as predictive factors for cardiac irAEs remains unclear, although these antibodies have been detected in several cases [40]. Collectively, large registries and cohorts are lacking for the assessment of the valid risk factors for ICI-related cardiotoxicity.

## 3. Pathogenic Mechanisms

The mechanism of ICI-related cardiotoxicity is not completely clear. Pathologically, ICI-associated myocarditis is associated with the infiltration of CD4+/CD8+ T cells, CD68+ macrophages, and a paucity of other immune cells in the myocardium [51]. Clonal cytotoxic Temra CD8+ cells are increased in the blood of patients with ICI myocarditis, as well as in the blood/hearts of Pdcd1-/- mice with myocarditis, and these CD8+ cells are featured in some unique transcriptional changes, including the upregulation of several chemokines such as CCL5 [60]. The direct T cell cytotoxic killing of cardiac cells and overexpressed proinflammatory cytokines by these activated immune cells might partly contribute to cardiac injury [61,62]. Spencer C. Wei demonstrated that CTLA4 in the context of the complete genetic absence of PD-1 leads to premature death featured in myocardial infiltration by T cells and macrophages and severe electrocardiographic abnormalities in mice, similar to what has been observed in ICI-associated myocarditis [63]. PD-1−/− BALB/c mice developed spontaneous dilated cardiomyopathy caused by auto-antibodies targeting cardiac troponin [64,65]. Correspondingly, in both CD8+ and CD4+ T cells mediated myocarditis models, a lack of PD-1 enhanced cardiac damage [66].

PD-1 deficiency in autoimmune murphy Roths Large (MRL) mice led to the development of fatal myocarditis with T cell and macrophage infiltration into the myocardium, and positive cardiac specific antibodies were detected [67]. Different B cell subtypes in the peripheral blood of cancer patients were found to be related to treatment response during ICI therapy [68], while on the other hand, they might also contribute to ICI-related irAEs. Apart from cardiac specific antibodies, autoantibodies induced by ICI therapy have also been detected, such as anti-acetylcholine receptors, anti-striated muscle antibodies, and anti-mitochondrial antibodies [69]. Anti-striated muscle antibodies, for example, might react with both cardiac and skeletal muscle antigens to induce antibody-dependent cellular cytotoxicity (ADCC) [70]. Further, autoantibodies could induce complement-dependent cytotoxicity (CDC) activation, leading to cell lysis [71]. These studies suggest that both cellular and humoral immunity contribute to ICI-related cardiotoxicity.

The mechanisms through which immune cells are recruited in the myocardium is incompletely understood. Immune checkpoints are not specifically expressed on tumor cells; instead, they are constitutively expressed in APCs, endothelial cells, cardiomyocytes, etc., providing potential targets for ICIs [72]. Alan H. Baik et al. proposed some other theories about the mechanisms of ICI-related cardiac irAEs, including the upregulation of autoantibodies targeting self-antigens, the systemic activation of T cells against normal cardiac tissues that bear the same antigens as tumor cells, and the release of subclinical or smoldering microbial-induced inflammation infiltration [73]. Clonal T cells targeting identical antigens in skeletal muscle and cardiac tissues might explain the concomitant presentation of myocarditis and myositis.

Few reports regarding inflammatory cytokines in ICI-related cardiac injury have been published. Several cytokines have been identified, including interferon γ, interleukin 1β (IL-1β), and the chemokine (C-X-C motif) ligand 10 (CXCL10) [69]. The release of several cytokines and chemokines is attributed to the recruitment of neutrophils and macrophages to the heart. In heart failure, increased levels of these cytokines lead to the local recruitment of T cells and other immune cells to further induce heart damage [74]. Cytokines such as IL-6 could directly increase the risk of cardiac arrhythmias [75]. Further, some cytokines could alter the excitation–contraction coupling to induce cardiac dysfunction by altering calcium handling [76]. IL-1β increases the risk of arrhythmias associated with CaMKII oxidation and phosphorylation in cardiomyocytes [77]. However, the precise role of cytokines in ICI induced myocarditis remains unknown.

## 4. Clinical Presentation

Several cardiotoxic irAEs have been reported, including myocarditis, pericardial disease, conduction abnormalities, congestive heart failure, Takotsubo-like syndrome, and acute coronary syndrome (ACS) [14,78]. ICI-related cardiotoxicity typically occurs early after ICI treatment. According to Escudier et al., the median time from treatment to the occurrence of presentations was 65 days after nearly three cycles of ICI treatment [24]. In another registry that enrolled 35 patients with ICI-associated myocarditis and 105 patients without myocarditis, the median time of onset was 34 days after starting ICI (inter-quartile range: 21 to 75 days) [14]. Sixty-four percent of myocarditis occurred after the first or second ICI dose, and 76% occurred within the first 6 weeks of treatment [15]. Some late-onset cases which occurred several months and years after ICI therapy have also been reported [79].

Patients may present with various unspecific symptoms including asymptomatic troponin elevation, fatigue, dyspnea, myalgia, palpitation, chest pain, lower extremity edema, headedness, syncope, and so on [14]. Severe cases may present with cardiogenic shock and even cardiac arrest. These symptoms occur in varied incidence, and 14–37% of patients with ICI-associated myocarditis may present with chest pain and 71–76% may present with dyspnea/fatigue [80]. It has been shown that approximately 10% of ICI-related cardiotoxicity cases developed cardiac conduction defects, and 5–10% of patients receiving ICI therapy developed ventricular arrhythmias, with a mortality rate of 40% [20]. ICI-related myocarditis is often combined with other organ damage such as diplopia (6%), myasthenia-like syndrome (11–30%), and myositis (23–30%) [80]. Due to the broad range of nonspecific clinical presentations, it is difficult to differentiate ICI-related cardiotoxicity with other cardiac conditions, such as acute coronary syndrome and heart failure, unless further rule-out tests are performed. Commonly used tests for ICI-related cardiac irAEs include an electrocardiogram (ECG), cardiac biomarkers, an echocardiogram, cardiac magnetic resonance (CMR) imaging, and an endomyocardial biopsy (EMB).

An ECG is a practical examination for the detection of ICI-related cardiotoxicity. Approximately 40–89% patients presented with an abnormal ECG, such as a prolonged PR interval, various arrhythmia, a declined R-wave amplitude, and a change of the ST-T segment [24]. These changes in ECG are commonly unspecific, and more tests are needed to establish the diagnosis of ICI-related cardiotoxicity. Cardiac biomarkers, including cardiac troponin I (TnI), creatine kinase (CK), creatine kinase myocardial band (CK-MB), brain natriuretic peptide (BNP), and N-terminal pro-brain natriuretic peptide (nt-proBNP) are efficient tools for assessing cardiac injury. TnI is now regarded as the most valuable biomarker for the diagnosis of ICI-related cardiotoxicity and is recommended to be tested during the first 6-week treatment of ICI for screening and surveillance [81,82]. An increase in troponin occurs in 46–94% of patients with ICI myocarditis, and an increase in NT-proBNP might be present in 65–100% of ICI myocarditis cases [80]. Echocardiography is an important tool for evaluating cardiac structure and function. Changed left LVEF, diastolic function, new wall motion abnormalities, and newly onset pericardial effusion, compared with baseline echocardiogram indexes, could suggest the diagnosis of ICI-related myocarditis [83]. Among patients with ICI-related myocarditis, echocardiographic global longitudinal strain (GLS) is reduced, and the decrease in GLS is associated with the incidence of major adverse events, which could be a potential indicator for the diagnosis and stratification of ICI-related myocarditis. CMR is now the most solid validation of myocarditis among the noninvasive cardiac imaging modalities. Different imaging techniques with CMR such as T2-weighted imaging, T 1/2 mapping, and late gadolinium enhancement (LGE) could demonstrate myocardial edema and injury. In a retrospective cohort study that enrolled patients with ICI myocarditis, myocardial T1 and T2 values were significantly elevated in 78% and 43% of patients, respectively, and T1 measurements were more significantly related with histopathological changes and were independent prognostic indicators for the subsequent development of MACE [84]. However, specific signs of CMR for ICI-related cardiotoxicity are unclear. Some patients with ICI-related myocarditis may show negative evidence of LGE, partly due to the early phase of the disease [24]. Therefore, repeated CMRs in 2 to 3 days should be considered when myocarditis is highly suspected. Endomyocardial biopsy is the gold standard for diagnosing ICI-related myocarditis. Inflammatory infiltrates and myocardial necrosis are typical in myocarditis [85]. The pathological classification of ICI myocarditis might also help to stratify the prognosis [86]. A skeletal muscle biopsy could be considered as an alternative for patients with suspected myocarditis and myositis if myocardial biopsy is not feasible [87]. In addition, coronary angiography is usually required to rule out coronary artery disease.

By integrating all the related clinical characteristics, uniform diagnostic criteria for ICI-related myocarditis were proposed in 2019 [88]. ICI-related myocarditis is categorized into definite myocarditis, probable myocarditis, and possible myocarditis [88]. A diagnosis of definite myocarditis can only be made if any of the following criteria are met: (1) cardiac biopsy indicates myocarditis; (2) myocarditis confirmed by CMR, together with elevated cardiac biomarkers or ECG evidence of myo-pericarditis; and (3) new wall motion abnormality on echocardiogram with no extra reasons, combined with typical clinical syndrome of myocarditis, elevated cardiac biomarkers, ECG evidence of myo-pericarditis, and negative tests to exclude obstructive coronary disease. According to The American Society of Clinical Oncology guidelines, ICI-related cardiotoxicity is ranked from grade 1 to 4, ranging from asymptomatic screening test results to life-threatening situations [89]. These guidelines help to instruct the clinical diagnosis and stratification of cardiac irAEs; however, due to the variety of ICI-related cardiotoxicities, some diagnostic criteria are hard to meet. More precise guidelines on the specific types of cardiac irAEs are necessary.

## 5. Baseline Screening and Surveillance

For the early recognition of ICI-related cardiac irAEs, a basic assessment on cardiac status, including ECG, echocardiography, and troponin I, should be considered before starting ICI therapy [88]. Low neutrophil-to-lymphocyte ratios (L-NLR) were associated with the risk of ICI-related cardiotoxicity; therefore, this may also serve as an indicator for cardiac irAEs [25]. During ICI treatment, it is recommended that all patients receive regular troponin I measurements every week [12]. If elevated troponin I is found, other tests such as an ECG, an echocardiography, a CMR, and even a cardiac biopsy should be carried out to further confirm the diagnosis of cardiac irAEs.

## 6. Treatment

Once a diagnosis of ICI-related cardiac irAEs is made, treatment must be initiated promptly to avoid severe adverse events. Until now, prospective studies and randomized trials assessing the efficacy of treatment for ICI-related cardiotoxicity have been lacking. Currently, the management of ICI-related cardiac irAEs focuses on immunosuppression therapy, together with ICI discontinuation. Generally, grade 3–4 toxicities require the initiation of high-dose corticosteroids (prednisone 1 to 2 mg/kg/d or methylprednisolone 1 to 2 mg/kg/d), and corticosteroids should be continued for at least 4 to 6 weeks [89]. A higher initial dose and an earlier initiation of corticosteroids have proven to be associated with improved outcomes [90]. Compared with low-dose corticosteroids (<60 mg/d), high doses (501–1000 mg/d) have been associated with a 73% lower risk of MACE, and patients receiving corticosteroids within 24 h of admission also had a lower rate of MACE (7.0%) than those receiving corticosteroids between 24 and 72 h (34.3%) [90].

Apart from corticosteroids, immunosuppressive therapies such as immunoglobulin, tacrolimus, infliximab, mycophenolate mofetil, and methotrexate might also serve as potential treatments for ICI-related myocarditis [91]. However, the efficacy of these second-line immunosuppressive has not been tested by large observational or prospective studies. The administration of over one of these agents, in addition to steroids, could be considered for patients who do not respond well to glucocorticoids [83]. Mariella Bockstahler demonstrated the potential of immunoproteasome inhibitors in improving heart-specific autoimmune responses, which could possibly benefit patients with ICI-related cardiac autoimmunity [92]. Therapeutic plasma exchange (TPE) was reported to be used in steroid-refractory, nivolumab-induced myocarditis, and fatal myocarditis combined with myositis [93,94]. Further, immunoadsorption therapy has been applied in dilated cardiomyopathy and heart transplantation to selectively remove immunoglobulins and immune complexes [95], which might be considered as a potential treatment for ICI-related irAEs when cardiac autoimmune antibodies are detected. Blocking tumor necrosis factor alpha (TNFα) has preserved left ventricular function in anti-PD1 treated melanoma, providing a potential approach to prevent ICI-induced cardiac injury [96]. The successful use of tocilizumab (an anti-CD52 antibody) and tocilizumab (an IL-6 receptor antagonist) on ICI-related myocarditis provides alternative options for corticosteroid-refractory cases.

Besides immunosuppressive treatment, conventional cardiac therapy should be initiated for patients with heart failure, ACS, and arrhythmias. These medications include β-blockers, angiotensin-converting enzyme inhibitors, aldosterone antagonists, etc. For ICI-related increases in aortic atherosclerotic plaque, concomitant statin and corticosteroid therapy could be applied. For patients with elevated hsTnI and cardiac conduction defects, it should be considered to transfer them to a coronary care unit. Pacemakers should be considered for patients with high-grade atrioventricular blocks [40]. Mechanical supports must be used for life-threatening cases.

Collectively, individualized treatment strategies made for patients require a multidisciplinary team composed of oncologists, cardiologists, and immunologists. However, ICI-related cardiotoxicity remains therapeutically challenging, and there is a lack of hard evidence to support these therapeutic strategies. There are limited data regarding the safety of re-introducing ICIs upon stabilization or the resolution of symptoms and/or laboratory abnormalities. Whether ICI therapy can be restarted remains challenging. ICI may be re-introduced in patients with grade 1–2 (but not grade 3–4) irAEs, with careful surveillance [97].

## 7. Other Immune Checkpoint Inhibitors

The past decade has witnessed several novel immune checkpoint inhibitors, apart from the CTLA-4, PD-1, and PD-L1 inhibitors. Lymphocyte activation gene-3 (LAG-3), an immunosuppressive molecule expressed on various immune cells (T cells, Tregs, B cells, natural killer cells, etc.), could increase the function of Tregs and inhibit CD8^+^ T cells [98]. Randomized controlled trials (RCT) regarding inhibitors of LAG-3 are ongoing, and some have shown positive effects of anti-LAG-3 therapy [6]. Opdualag, a compound preparation of relatlimab (anti-LAG-3) and nivolumab (anti-PD-1), has recently been approved by the FDA for the treatment of previously untreated metastatic or unresectable melanoma. T cell immunoglobulin and mucin-domain containing-3 (TIM-3) is another target for cancer therapy. By interacting with its ligand, TIM-3 could diminish anti-tumor effects partly by inducing CD8^+^ T cell exhaustion [99]. RCTs on antibodies against TIM-3 have also been launched and a promising efficacy has been reported [6]. The T cell immunoglobulin and ITIM domain (TIGIT) is an immune checkpoint molecule that induces immunosuppressive activity in DCs and NK cells [100,101]. Clinical trials (NCT03563716) proved the efficacy of Tiragolumab (an anti-TIGIT) combined with atezolizumab (a PD-L1 antibody) in metastatic NSCLC patients. Other inhibitory checkpoint molecules that could serve as potential therapeutic targets include VISTA, CD276, CD272, and sialic acid-binding immunoglobulin-like lectin 15 (Siglec-15) [6].

In addition, immune checkpoints for positive immune regulation have also been introduced for cancer therapy. An agonist-targeting immune co-stimulator (ICOS) that enhances the function of CD8+ T cells and Tregs is now being studied (NCT02904226, NCT02723955, and NCT03251924). Several agonist antibodies for the glucocorticoid-induced TNFR-related gene (GITR) and OX40, molecules that belong to the tumor necrosis factor (TNF) receptor superfamily, have also been developed (NCT02598960, NCT02410512, etc.).

These novel immune checkpoint inhibitors and agonists provide promising options for cancer therapy. However, whether these treatments could lead to cardiac irAEs should be carefully monitored once they are applied in clinical work.

## 8. Conclusions

ICI treatment has revolutionized cancer therapy and improved the long-term survival of cancer patients. Along with the prosperity of ICIs, irAEs have emerged as a main obstruction to prevent patients from gaining clinical benefits. ICI-related cardiotoxicity is one of the rare but fatal irAEs that have been underestimated. Efforts have been made to elucidate the mechanisms of ICI-related cardiotoxicity, and diagnostic approaches for early identification have also been developed. However, the mechanisms through which ICIs lead to cardiac irAEs remains incompletely understood. Specific and noninvasive tests for the diagnosis of ICI-related cardiac toxicity are still lacking. Prospective and large RCTs assessing the efficacy of treatments on ICI-induced cardiac injury are in great demand. Future studies are warranted to enable a better understanding of the mechanisms underlying ICI-related toxicity and to facilitate the diagnostic, prognostic, and therapeutic strategies to improve the outcomes of cancer patients.

## Figures and Tables

**Table 1:** Marketed ICIs worldwide.

Immune Checkpoint Inhibitors	Target	Approval Year	Indications	Reported Cardiac irAEs
Ipilimumab	CTLA-4	2011 (FDA)	Melanoma, renal cell carcinoma, and colorectal cancer	Myocarditis [14], Takotsubo syndrome [27], pericardial diseases [11], and arrhythmia [28]
Nivolumab	PD-1	2014 (FDA)	Melanoma, renal cell carcinoma, non-small-cell lung cancer, head and neck squamous cell cancer, Hodgkin’s lymphoma, colorectal cancer, urothelial carcinoma, and hepatocellular carcinoma	Myocarditis [14], Takotsubo syndrome [27], heart failure [29], pericardial diseases [11], and arrhythmias [30]
Pembrolizumab	PD-1	2014 (FDA)	Melanoma, small-cell lung cancer, non-small-cell lung cancer, head and neck squamous cell cancer, Hodgkin’s lymphoma, colorectal cancer, urothelial carcinoma, hepatocellular carcinoma, large B-cell lymphoma, gastric cancer, esophageal cancer, cervical cancer, renal cell carcinoma, and Merket cell carcinoma	Myocarditis [14], Takotsubo syndrome [27], and pericardial diseases [11]
Atezolizumab	PD-L1	2016 (FDA)	Urothelial carcinoma and non-small-cell lung cancer	Myocarditis [14], pericardial diseases [11], and hypertension [31]
Avelumab	PD-L1	2017 (FDA)	Merket cell carcinoma	Myocarditis [11] and pericardial diseases [11]
Durvalumab	PD-L1	2017 (FDA)	Urothelial carcinoma	Myocarditis [11] and pericardial diseases [11]
Dstarlimab	PD-1	2021 (FDA)	MSI-H/dMMR advanced solid tumors	N/A
Cemiplimab	PD-1	2018 (FDA)	Cutaneous squamous cell carcinoma	Myocarditis [32] and hypertension [33]
Toripalimab	PD-1	2018 (NMPA)	Melanoma, nasopharyngeal carcinoma, urothelial carcinoma, esophageal squamous cell carcinoma, and non-small cell lung cancer	N/A
Sintilimab	PD-1	2018 (NMPA)	Hodgkin’s lymphoma, non-small cell lung cancer, and hepatocellular carcinoma	Myocarditis [34], arrhythmia [35], and heart failure [36]
Camrelizumab	PD-1	2020 (NMPA)	Hodgkin’s lymphoma, hepatocellular carcinoma, non-small cell lung cancer, esophageal squamous cell carcinoma, and nasopharyngeal carcinoma	Myocarditis [37], heart failure [38], arrhythmia [38], and coronary artery spasm [39]
Tislelizumab	PD-1	2019 (NMPA)	Hodgkin’s lymphoma, urothelial carcinoma, non-small cell lung cancer, hepatocellular carcinoma, esophageal squamous cell carcinoma, nasopharyngeal carcinoma, and MSI-H/dMMR advanced solid tumors	Myocarditis [40] and arrhythmia [40]
Prolgolimab	PD-1	2020 (MHRF)	Melanoma	N/A
Sugemalimab	PD-L1	2021 (NMPA)	Non-small cell lung cancer	Heart failure [41]
Penpulimab	PD-1	2021 (NMPA)	Hodgkin’s lymphoma	N/A
Zimberelimab	PD-1	2021 (NMPA)	Hodgkin’s lymphoma	N/A
Envafolimab	PD-L1	2021 (NMPA)	MSI-H/dMMR advanced solid tumors	N/A
Serplulimab	PD-1	2021 (NMPA)	MSI-H/dMMR advanced solid tumors	N/A

CTLA-4, cytotoxic T lymphocyte-associated antigen-4; FDA, Food and Drug Administration; irAEs, immune-related adverse effects; MHRF, Ministry of Health of the Russian Federation; NMPA, National Medical Products Administration; MSI-H/dMMR, microsatellite instability-high/mismatch repair deficient; PD-1, programmed cell death protein 1; PD-L1, programmed cell death ligand 1; N/A, not available.

**Table 2:** The prime publications about cardiotoxicity related to immune checkpoint inhibitors from 2018–2022.

References	Study Type	Research Period	Sample Size	Data Source	Types of Cancer	Cardiac irAEs (n/%)	Mortality of Cardiac irAEs (n/%)	Different Types of Cardiac irAEs					
								Myocarditis (n/%)	Pericardial Disease (n/%)	Arrhythmia (n/%)	Myocardial Infarction (n/%)	Heart Failure (n/%)	Cardiac Arrest (n/%)
Wang D.Y. et al. [10]	Meta-analysis	From 2009 to 2018	613	WHO pharmacovigilance database	Melanoma, lung cancer, and other	N/A	52/39.7	131/21.4	N/A	N/A	N/A	N/A	N/A
Mahmood S.S. et al. [14]	Retrospective study	From December 2013 to July 2017	35	The electronic medical records from eight centers	Multiple cancer types	N/A	16/45.7	35/100	N/A	N/A	N/A	N/A	6/17.1
Chitturi K.R. et al. [42]	Retrospective study	From August 2015 to August 2018	135	Houston Methodist oncologic pharmacy registry	Lung cancer	18/13.3	13/9.6	1/0.7	9/6.7	25/18.5	1/0.7	4/3.0	N/A
Awadalla M. et al. [43]	Retrospective study	From December 2013 to January 2019	101	19-center international registry	Multiple cancer types	101/100	6/5.9	101/100	N/A	19/18.8	N/A	N/A	12/11.9
Moey M.Y.Y. et al. [44]	Retrospective study	From 2015 to 2018	196	Vidant Medical Center/East Carolina University	Lung cancer	23/11.0	3/1.5	9/4.6	4/2.0	7/3.6	3/1.5	N/A	N/A
Agostinetto E. et al. [45]	Meta-analysis	Prior to 30 June 2020	6092	PubMed, MEDLINE, Embase databases, and conference proceedings	Multiple cancer types	230/3.78	55/0.33	16/0.12	31/0.51	104/1.79	27/0.41	28/0.43	19/0.24
Rubio-Infante N. et al. [46]	Meta-analysis	Prior to 31 August 2020	104,276	WHO’s Vigi Access database	N/A	4401/4.2	15/0.32	839/19.1	335/7.6	850/19.3	287/6.5	348/7.9	257/5.8
Chen C. et al. [47]	Pharmacovigilance study	From 2014 to 2019	9271	FDA Adverse Event Reporting System database	N/A	N/A	2808/30.3	614/16	423/3.6	576/4.8	N/A	476/4.0	N/A
Mascolo A. et al. [48]	Retrospective study	Prior to 14 March 2020	2478	European pharmacovigilance database	N/A	N/A	N/A	542/16	229/6.8	221/6.5	166/4.7	242/7.1	107/3.2
Li C. et al [49]	Retrospective study	Prior to 17 February 2022	5518	Health care organizations in the research network of TriNetX	Multiple cancer types	690/12.5	N/A	116/2.1	N/A	513/9.3	N/A	N/A	N/A
Chan J.S.K. et al. [26]	Retrospective study	From 1 January 2013 to 31 December 2021	4324	A population-based, administrative electronic medical records system in Hong Kong	Multiple cancer types	188/4.4	N/A	N/A	N/A	97/2.2	46/1.1	52/1.2	N/A

N/A, not available. Despite increased reporting of ICI-related cardiotoxicity, the underreporting of potential cases has drawn wide attention. Incidence rates calculated from clinical trials, but not from spontaneous reporting, could be misleading [50]. This may partly explain the varied range of incidence and fatality rate of ICI-related cardiotoxicity reported by current studies. Therefore, standardized reporting of cardiac adverse events is required in clinical trials.
